# The impact of chronic kidney disease on developed countries from a health economics perspective: A systematic scoping review

**DOI:** 10.1371/journal.pone.0230512

**Published:** 2020-03-24

**Authors:** Sarah Elshahat, Paul Cockwell, Alexander P. Maxwell, Matthew Griffin, Timothy O’Brien, Ciaran O’Neill

**Affiliations:** 1 Centre for Public Health, Queen’s University Belfast, Belfast, Northern Ireland, United Kingdom; 2 University Hospitals Birmingham, Birmingham, England, United Kingdom; 3 National University of Ireland Galway, Galway, Ireland; Universidade Estadual Paulista Julio de Mesquita Filho, BRAZIL

## Abstract

Chronic kidney disease (CKD) affects over 10% of the global population and poses significant challenges for societies and health care systems worldwide. To illustrate these challenges and inform cost-effectiveness analyses, we undertook a comprehensive systematic scoping review that explored costs, health-related quality of life (HRQoL) and life expectancy (LE) amongst individuals with CKD. Costs were examined from a health system and societal perspective, and HRQoL was assessed from a societal and patient perspective. Papers published in English from 2015 onward found through a systematic search strategy formed the basis of the review. All costs were adjusted for inflation and expressed in US$ after correcting for purchasing power parity. From the health system perspective, progression from CKD stages 1–2 to CKD stages 3a-3b was associated with a 1.1–1.7 fold increase in per patient mean annual health care cost. The progression from CKD stage 3 to CKD stages 4–5 was associated with a 1.3–4.2 fold increase in costs, with the highest costs associated with end-stage renal disease at $20,110 to $100,593 per patient. Mean EuroQol-5D index scores ranged from 0.80 to 0.86 for CKD stages 1–3, and decreased to 0.73–0.79 for CKD stages 4–5. For treatment with renal replacement therapy, transplant recipients incurred lower costs and demonstrated higher HRQoL scores with longer LE compared to dialysis patients. The study has provided a comprehensive updated overview of the burden associated with different CKD stages and renal replacement therapy modalities across developed countries. These data will be useful for the assessment of new renal services/therapies in terms of cost-effectiveness.

## 1. Introduction

Chronic kidney disease (CKD) is a major global public health issue, affecting over 10% of the population worldwide [1]. The problem was ranked 16^th^ among the leading causes of death in 2016, and is expected to rise to 5^th^ ranked by 2040 [2]. Chronic kidney disease is defined as an abnormality of kidney function or structure for ≥ 3 months [3] and is a significant burden for individuals, health care systems and societies; it is associated with increased hospitalization, productivity loss, morbidity and early mortality. Diabetic kidney disease is the leading cause of CKD [4]; other common causes include hypertension, glomerulonephritis and autosomal dominant polycystic kidney disease [5]. Many individuals have no known cause of CKD.

Chronic kidney disease is classified based on abnormal urinalysis and/or renal tract structure and estimated glomerular filtration rate (eGFR), with the most advanced stage, CKD stage 5, comprising individuals with an eGFR <15 ml/min/1.73m^2^ including patients with end-stage renal disease (ESRD) [1]. Chronic kidney disease is managed through treatment of its risk factors, such as hypertension and diabetes mellitus [3]. A small proportion of patients with CKD progress to ESRD requiring renal replacement therapy (RRT) with dialysis and/or kidney transplantation (KTx) [6]. Dialysis treatment can be provided at home (peritoneal dialysis (PD) or home haemodialysis (HHD)), or as in-centre haemodialysis (ICHD) at a satellite dialysis unit or hospital. [7]. Conservative care is an alternate to RRT for patients who would not have a survival or quality of life benefit from RRT or who choose not to receive RRT, and only provides a means for management of symptoms associated with CKD [8]. In 2010, approximately 2.62 million patients received RRT worldwide, and the numbers are expected to double by 2030, in part due to trends in obesity and diabetes mellitus and in part due to an ageing population in many countries [9]. The increasing incidence of ESRD treatment can also be linked to the improved access to ESRD services in developed countries [10].

In 2016, after Taiwan, the US showed the second highest incidence rate of RRT worldwide, with 378 patients per million of the general American population receiving treatment for ESRD [11].

The current scoping review aimed to provide an updated comprehensive summary of the reported/estimated burden associated with CKD and different RRT modalities in order to inform cost-effectiveness analyses of novel renal services/therapies. Specific objectives of this study were (1) to investigate the costs of CKD and/or RRT from a health system and societal perspective, (2) to explore HRQoL among CKD patients receiving or not receiving RRT, from a societal and patient perspective, and (3) investigate LE and survival in patients with CKD receiving or not receiving RRT.

## 2. Methodology

A systematic scoping review was undertaken to assess the nature, scope, and range of the existing literature on the study topic, and to produce a comprehensive synthesis of the available evidence. S1 Appendix shows the PRISMA checklist for this study. A systematic scoping review was chosen because of the potential breadth of the literature to be examined and the desire to identify gaps in knowledge. Systematic scoping reviews merge the advantages of the scoping review methodology (e.g. implementation of consultation exercise with key stakeholders to validate findings) and the strengths of the systematic review method (e.g. adoption of systematic search strategy). This study adopted the six-stage methodological framework set by Arksey and O’Malley [12] as follows:

### 2.1. Setting the research question

In order to comprehensively review the literature, a broad research question was formulated: *what are the associated societal costs of CKD and what are the HRQoL and the LE of patients with CKD*?

### 2.2. Identification of pertinent studies

A flexible search strategy was adopted to find relevant published citations and grey literature (e.g. government documents and reports) on the study topic. Four key concepts were identified in the review question, namely, *CKD*, *societal costs*, *HRQoL and LE*–each being seen as a facet of disease burden. In total, 24 search terms were operated using three separate combinations utilizing Boolean operators ‘AND’ and ‘OR’ (S2 Appendix).

The search technique included both automated and manual search methods in order to avoid missing any pertinent articles. The automated search was undertaken by one author (SE) in five databases, namely, Medline, Embase, the National Health Service Economic Evaluation Database, the Health Technology Assessment Database, and the Database of Abstracts of Reviews of Effects. Given the periodic revision and update of international CKD care guidelines, the search process was limited to articles published between 2015 and April 2019 to provide a comprehensive updated overview of the burden associated with the disease, while aiding comparability. [13] We limited the search to this five-year period in order to extract cost estimates from a limited, and therefore in economic terms, comparable time period.

The search strategy also included searching bibliographies of potential articles, hand searching journals, as well as researching key authors, conference abstract repositories, and institutional websites.

### 2.3. Study selection

The study only included English language articles published during the search window (2015–2019). In an attempt to strike a balance between minimising heterogeneity while being comprehensive, only studies conducted in North America (Canada and US), Europe, and Australia were included. Studies were included if they estimated health care costs and/or total societal costs (lost productivity and out of pocket costs, including informal care, paid domestic help and transportation), HRQoL and/or the LE of adult patients with CKD. Articles assessing utility values, without assessing HRQoL scores were included. The following study types were excluded: (1) studies investigating patients’ perceptions about RRT; (2) studies exploring HRQoL among carers/family members of CKD patients; and (3) studies concerned with incremental costs without estimating actual costs (e.g. the cost of a service rather than just the cost of an addition to that service).

**Fig 1:**
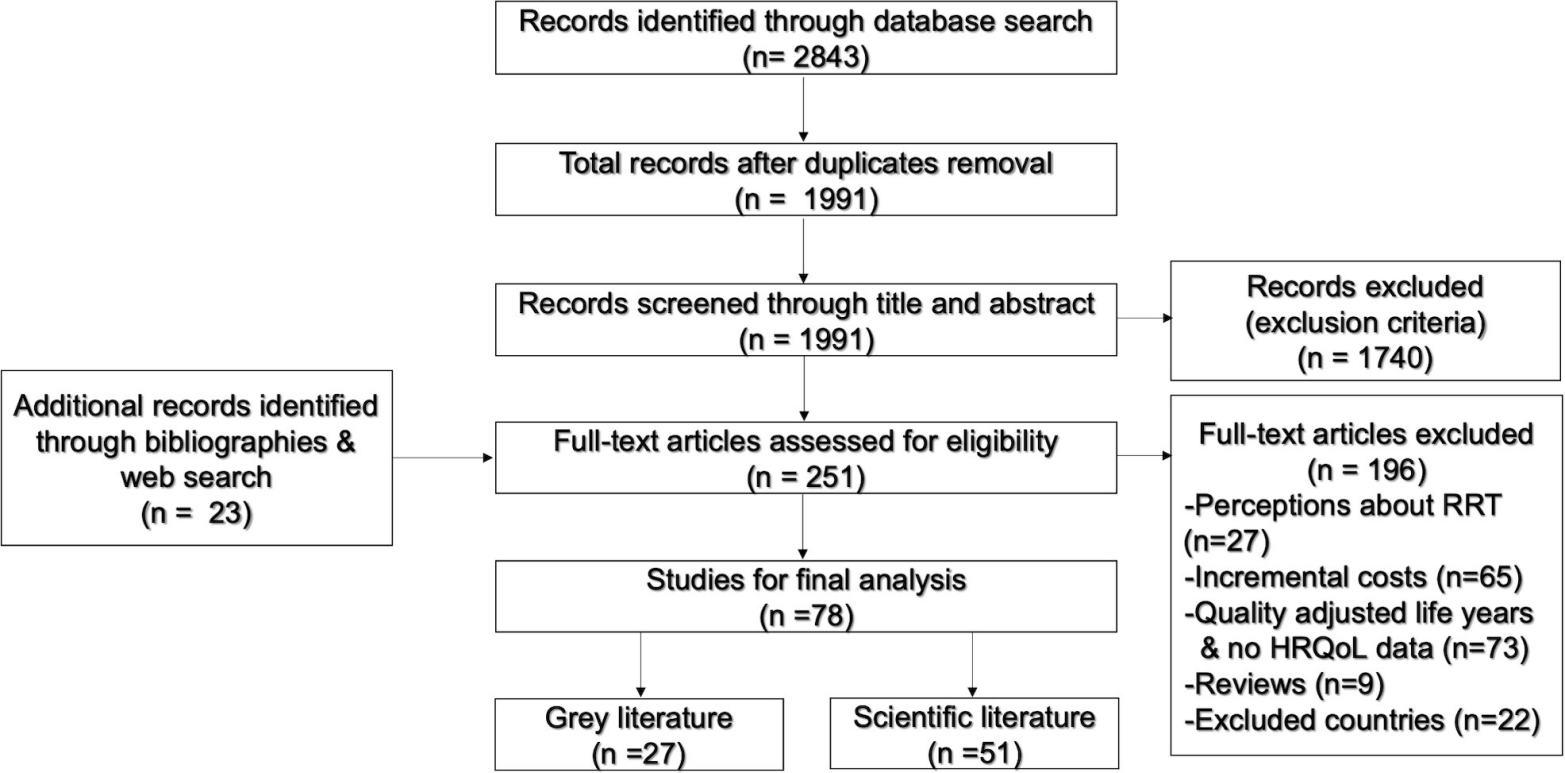
Flow diagram of the studies included in the final analysis.

A two-step process was followed to select eligible studies from among the search results. First, titles and abstracts of the retrieved articles were preliminarily screened against the inclusion and exclusion criteria. Second, the potentially relevant articles were re-examined and reviewed in full, together with the papers located from reference lists and web searches (Fig 1).

### 2.4. Charting the data

Standardized Microsoft Excel extraction forms were used to extract pertinent data from the included articles. These incorporated authors’ names, article title, country of authorship, study objectives, study methodology, study participants, and main findings. With respect to the reporting of costs, to aid comparability, the study adjusted for inflation and purchasing power parity using a two-step process. Costs in the included studies were first adjusted for inflation in their country of origin using the relevant consumer price index for health to express these in a common year’s values and then adjusted for purchasing power parity using Organisation for Economic Co-operation and Development (OECD) published data [14,15]. All costs were expressed in US$ 2019.

### 2.5. Collating, summarizing and reporting the results

The data analysis phase comprised two major steps in order to reliably categorize complex data, and to answer the research question in such a way that the outputs were consistent with the study aim and objectives. First, the extracted data were subjected to numerical synthesis in order to map the available evidence as outlined in the review question. Second, the data were distributed among three major subsections addressing the study objectives separately, while considering the implications of the study findings to improve the validity of the study and to inform future research.

### 2.6. Consultation exercise

The study included a consultation with four nephrologists from the UK and Ireland. This involved the circulation of a preliminary copy of the review (i.e. aims, objectives, search strategy and major findings) together with a brief survey to explore their opinions on its findings (S3 Appendix). The aim of the survey was to validate the study findings. The consultation output was taken into consideration in the subsequent discussion of the study.

## 3. Results

In total, 1991 potential papers were retrieved from the searched databases, with an additional 24 located from web searches and bibliographies. Of these, only 78 were ultimately included in the review (Fig 1). The majority of the included studies were conducted in Europe (62%), followed by North America (31%), and Australia (7%). Two study designs predominated: cross-sectional study (38%) and cohort study (33%) (S1 Table).

### 3.1. Costs of CKD

A total of 37 studies estimating health care and societal costs of CKD and RRT were included. These investigated costs associated with different stages of CKD, and costs of different treatment modalities as follows:

#### 3.1.1. Costs of different CKD stages

The annual societal and health care costs increased with the progression of each CKD stage, regardless of the cause of CKD or the country of origin (Tables 1 and 2).

**Table 1:** Per patient mean annual total health care costs (in 2019 US $) for different CKD stages or ESRD (Mean ± SD / (95% CI))^&^.

Country	Study type	Participants		Any CKD	CKD 1	CKD 2	CKD 3a	CKD 3b	CKD 4	CKD 5	ESRD	Reference
US	Retrospective cohort study	250,742 DKD patients		40,259^δ^	N/A	N/A	N/A	N/A	N/A	N/A	N/A	[16]
US	Retrospective cohort study	1,274 ADPKD patients with ESRD		N/A	N/A	N/A	N/A	N/A	N/A	N/A	64,040^#^	[17]
Sweden	Prospective cohort study.	2432 Patients with CKD		N/A	N/A	N/A	N/A	N/A	12,200*		N/A	[18]
Denmark	Cross-sectional	243 ADPKD patients		N/A	3,675 (1,931–6,666) ^#^*				6,190 (4,703–8,392) ^#^*		N/A	[19]
Norway	Cross-sectional	243 ADPKD patients		N/A	3,865 (1,870–6,930) ^#^*				8,009 (5,112–11,846) ^#^*		N/A	[19]
Sweden	Cross-sectional	243 ADPKD patients		N/A	3,181 (1,780–5,595) ^#^*				5,367 (4,053–7,192) ^#^*		N/A	[19]
Finland	Cross-sectional	243 ADPKD patients		N/A	4,417 (2,671–7,438) ^#^*				7,070 (5,513–9,254) ^#^*		N/A	[19]
US	Case-control study	106,050 patients aged ≤65 with CKD		N/A	N/A	16,692	24,147	31,596	53,186		100,594	[20]
Italy	Prospective cohort study	864 patients with CKD		3,599 (3,255–3,942)	1,545 (1,147–1,944)	1,990 (1,604–2,376)	2,161 (1,797–2,524)	3,377 (2,783–3,970)	5,481 (4,472–6,489)	7,207 (5,639–8,773)	N/A	[21]
UK	Secondary analysis of the SHARP data	7,246 CKD patients		N/A	1,600^+^				5,604^+^	19,648^+^	N/A	[22]
England	Analysis of NHSRC data	211,215 CKD patients		16,377^+^	N/A	N/A	N/A	N/A	N/A	N/A	N/A	[23]
US	Retrospective cohort study	4234 ADPKD patients with CKD		N/A	26,367^#^^∝^	21,761^#^^∝^	26,982^#^^∝^		39,183 ^#^^∝^	N/A	N/A	[24]
US	Cross- sectional	18,380 ESRD patients aged ≥18 years		N/A	N/A	N/A	N/A	N/A	N/A	N/A	54,782± 132,992	[25]
Canada	Cohort study	219,641 patients with CKD		11,064	N/A	N/A	9,668	15,215	17,618	33,202	N/A	[26]
US	Cross-sectional	2,053 patients with DKD		25,150 (19,806–30,495)^δ^	N/A	N/A	N/A	N/A	N/A	N/A	N/A	[27]
US	Analysis of MEPS-HC	187,341 patients aged ≥18		44,699 (38,896–50,501)	N/A	N/A	N/A	N/A	N/A	N/A	N/A	[28,29]
Italy	Retrospective cohort study	130,502 DKD patients		N/A	N/A	N/A	N/A	N/A	N/A	N/A	20,110± 21,433^δ^	[30]
Italy	Retrospective cohort study	1067 CKD & ESRD patients		N/A	N/A		3,080± 4,518		N/A		N/A	[31]
Italy	Cross-sectional	484 patients with CKD		N/A	N/A	N/A	N/A	N/A	5,217± 5,491	6,859± 5,457	N/A	[32]
				N/A	N/A	N/A	N/A	N/A	7,587^δ^		N/A	
US	Medicare data analysis	Patients aged ≥65 with CKD^Ω^		23,036	20,104		22,318		30,080		N/A	[33]
				26,194^δ^	22,640^δ^		25,683^δ^		34,073^δ^		N/A	
US	Medicare data analysis	Patients aged ≥65 with CKD^Ω^		22,493	19,847		22,054		29,448		N/A	[34]
				25,015^δ^	22,701^δ^		25,280^δ^		32,853^δ^		N/A	
The Netherlands	Case-control study	18,340 patients with CKD 4/5	20–44 years	N/A	N/A	N/A	N/A	N/A	10,098		N/A	[35]
			45–64 years	N/A	N/A	N/A	N/A	N/A	13,539		N/A	
			65–74 years	N/A	N/A	N/A	N/A	N/A	15,172		N/A	
			≥75 years	N/A	N/A	N/A	N/A	N/A	13,958		N/A	
Australia	Cohort study	6138 patients with CKD		N/A	2,273 (1,945–2,600) *		2,916 (2,305–3,528) *		12,157 (4,747–37,480) *		N/A	[36]

^**&**^ Most studies estimated total health care costs, irrespective whether it is or is not related to CKD, based on primary care, in-patient, out-patient, diagnostics and medications/pharmaceuticals with exception of the following;

^+^ Studies estimated health care costs based on in-patient costs.

* Studies estimated health care costs solely related to CKD.

Cells are merged for studies which estimated combined cost for stages 1 &2, 1–3, 3a &3b, and 4 &5. Abbreviations: SD: standard deviation; CI: confidence interval; CKD: chronic kidney disease; DKD: diabetic kidney disease; ADPKD: autosomal dominant polycystic kidney disease; ESRD: end-stage renal disease; SHARP: The Study of Heart and Renal Protection; NHSRC: National Health Service Reference Cost; MEPS-HC: The Medical Expenditure Panel Survey Household Component; N/A: non-available.

^#^ Costs in patients with ADPKD.

^δ^Costs in patients with DKD.

^∝^Costs were extrapolated from biannual or monthly costs.

^Ω^ Number of patients is non-available.

**Table 2:** Mean annual societal costs & productivity loss (in 2019 US $) incurred by patients with CKD and on RRT (Mean ± SD / (95% CI)).

Country	Cost & productivity loss	CKD 1–3	CKD 4	CKD 5	Dialysis	KTx^@^	Reference
Denmark	**Societal cost**	10,431 (7,190–14,321) ^#^	18,600 (13,848–23,795) ^#^^δ^		100,758 (92,754–108,954) ^#^	36,430 (29,801–43,672) ^#^	[19]
	**Productivity loss**	6,717 (4,175–9,879) ^Φ^	12,076 (8,272–16,202) ^#^^δ^^Φ^		11,982 (7,792–16,020) ^Φ^	10,576 (7,142–14,360) ^Φ^	
Finland	**Societal cost**	10,261 (7,261–13,977) ^#^	18,971 (14,428–24,044) ^#^^δ^		87,106 (80,577–94,964) ^#^	33,515 (27,241–40,524) ^#^	[19]
	**Productivity loss**	5,810 (3,556–8,537) ^Φ^	11,617 (7,914–15,644) ^#^^δ^^Φ^		8,151 (5,511–10,593) ^Φ^	8,998 (6,078–12,476) ^Φ^	
Norway	**Societal cost**	15,001(10,469–20,282) ^#^	28,428 (20,654–37,285) ^#^^δ^		106,614 (94,981–120,328) ^#^	36,702 (27,876–46,595) ^#^	[19]
	**Productivity loss**	11,082 (7,004–15,737) ^Φ^	19,951 (13,970–26,285) ^#^^δ^^Φ^		19,783 (12,969–25,893) ^Φ^	18,013 (12,334–24,087) ^Φ^	
Sweden	**Societal cost**	10,274 (7,148–13,893) ^#^	19,556 (14,476–25,072) ^#^^δ^		92,440 (85,156–99,702) ^#^	31,557 (25,169–38,880) ^#^	[19]
	**Productivity loss**	7,060 (4,354–10,155) ^Φ^	13,910 (9,729–18,319) ^#^^δ^^Φ^		12,745 (8,501–16,609) ^Φ^	12,325 (8,367–16,494) ^Φ^	
Italy	**Societal cost**	N/A	9,734± 8,204	11,766± 8,529^δ^	N/A	N/A	[32]
	**Productivity loss**	N/A	489± 2811*	1,045± 4,324^δ^*	N/A	N/A	
Australia	**Societal cost**	4,803	17,488^δ^		N/A	N/A	[37]
	**Productivity loss**	N/A	N/A		N/A	N/A	
Nordics	**Societal cost**	11,920^#^	20,142^#^^δ^		88,943^#^	37,849^#^	[38]
	**Productivity loss**	N/A	N/A		N/A	N/A	

Productivity losses in all studies were estimated using the human capital approach. All studies estimated productivity loss for all patients aged ≥18 years, including those over 65 years. In all studies, lost productivity was estimated among all patients in and not in the workforce. Societal costs in all studies were based on total health care costs, productivity loss, out of pocket costs, such as informal care and paid domestic help. Cells are merged for studies which estimated combined cost for stages 4 & 5. Abbreviations: SD: standard deviation; CI: confidence interval; CKD: chronic kidney disease; RRT: renal replacement therapy; KTx: kidney transplantation; N/A: non-available.

^@^ Combined KTx costs for those in the first or subsequent years.

^#^ Costs in patients with ADPKD.

^δ^ Cost in patients without dialysis.

* Productivity costs were estimated among patients and care givers.

^Φ^ Productivity loss was estimated in CKD patients only, with no estimation for care givers.

From a health system perspective, the per patient mean annual total health care costs ranged from $1,600 to $25,037 for patients with CKD stages 1–3, whereas patients with CKD stages 4–5 incurred higher costs ranging from $5,367 to $53,186. ESRD costs were even higher, ranging from $20,110 to $100,593. From a societal perspective, non-dialysis costs of CKD stages 1–3 were in the range $4,803-$15,001, whereas CKD stages 4–5 showed the highest costs ranging from $10,750 to $28,428 in Europe (Table 2). Turchetti et al., and Erikson et al., showed that productivity loss for patients with CKD stages 4–5 accounted for <1/10 and 2/3 of the total societal costs in Italy and the Nordic countries, respectively [19,32].

#### 3.1.2. Costs of different treatment modalities for ESRD

Eighteen studies investigated annual costs of different dialysis modalities, from a health system perspective (Table 3).

**Table 3:** Mean annual health care costs (in 2019 US $) of different dialysis modalities and kidney transplantation (Mean ± SD/ (95% CI)).

Country	Study type	Participants		KTx^@^	1^st^ year after KTx	Subsequent years after KTx	Any dialysis	Conventional HD		PD			References
								HHD	ICHD	Any PD	APD	CAPD	
Sweden	Cohort study	2432 Patients on RRT		19,698	N/A	N/A	N/A	N/A	111,326	74,471	N/A	N/A	[18]
Denmark	Cross-sectional	243 ADPKD patients		25,718 (20,858–31,208) ^#^	N/A	N/A	87,517 (81,751–94,503) ^#^	N/A	N/A	N/A	N/A	N/A	[19]
Finland	Cross-sectional	243 ADPKD patients		24,400 (19,501–30,242) ^#^	N/A	N/A	77,883 (72,654–84,620) ^#^	N/A	N/A	N/A	N/A	N/A	[19]
Norway	Cross-sectional	243 ADPKD patients		18,498 (13,183–25,139) ^#^	N/A	N/A	85,070 (76,481–95,863) ^#^	N/A	N/A	N/A	N/A	N/A	[19]
Sweden	Cross-sectional	243 ADPKD patients		19,118 (14,994–24,082) ^#^	N/A	N/A	78,646 (73,743–84,612) ^#^	N/A	N/A	N/A	N/A	N/A	[19]
UK	Analysis of SHARP data	7,246 CKD and ESRD patients		N/A	37,321 (36,449–38,195) ^+^	1,742 (1,484–1,999) ^+^	35,385 (35,241–35,529) ^+^	N/A	N/A	N/A	N/A	N/A	[22]
US	Cohort study	4,234 ADPKD patients on RRT		80,876^#^^∝^	N/A	N/A	145,215^#^^∝^	N/A	N/A	N/A	N/A	N/A	[24]
The Netherlands	Case-control study	13,734 patients on RRT	20–44 yrs	18,157	N/A	N/A	105,293	N/A	105,579	94,986	N/A	N/A	[35]
			45–64 yrs	18,219	N/A	N/A	110,123	N/A	111,275	97,796	N/A	N/A	
			65–74 yrs	20,472	N/A	N/A	107,443	N/A	108,205	96,654	N/A	N/A	
			≥75 yrs	19,387	N/A	N/A	100,215	N/A	100,668	92,767	N/A	N/A	
Nordics	Cross-sectional	243 ADPKD patients		21,358 ^#^	N/A	N/A	72,719 ^#^	N/A	N/A	N/A	N/A	N/A	[37]
Canada	Cost minimiza-tion study	Patients on RRT ^Ω^		N/A	N/A	N/A	N/A	31,734	49,071	29,542	N/A	N/A	[38]
France	Simulati-on model covering 15 years	Incident ESRD patients ^Ω^	18–44 yrs	N/A	N/A	N/A	N/A	N/A	N/A	N/A	35,020	35,100	[39]
				N/A	N/A	N/A	N/A	N/A	N/A	N/A	59,529^δ^	58,954^δ^	
			45–69 yrs	N/A	N/A	N/A	N/A	N/A	N/A	N/A	47,976	47,883	
				N/A	N/A	N/A	N/A	N/A	N/A	N/A	80,081^δ^	80,041^δ^	
France	Analysis of FNHIF data	47,862 ESRD patients ≥18 years		14,067^∝^	N/A	N/A	N/A	52,416^∝^	92,690^∝^	N/A	56,240^∝^	46,947^∝^	[40]
				23,199^δ^^∝^	N/A	N/A	N/A	66,188^δ^^∝^	107,078^δ^^∝^	N/A	68,983^δ^^∝^	56,922^δ^^∝^	
Italy	Clinical trial	340 patients with ESRD		N/A	N/A	N/A	N/A	N/A	71,191	N/A	N/A	N/A	[41]
Sweden	Before- after study^¥^	1220 ESRD patients		N/A	73,661±86,236	13,667	N/A	N/A	N/A	N/A	N/A	N/A	[42]
England	Analysis of UKRR data	20,235 patients on RRT		N/A	12,266^Φ^^%^	3,980^Φ^^%^	N/A	N/A	9,646^Φ^^%^	9,009^Φ^^%^	N/A	N/A	[43]
				N/A	15,381^δ^ ^Φ^^%^	N/A	N/A	N/A	14,436^δ^^Φ^^%^	N/A	N/A	N/A	
UK	Cross-sectional	Patients on RRT ^Ω^		21,534	N/A	N/A	N/A	14,616	29,665	16,067	N/A	N/A	[44]
US	Cohort study	181 ESRD immigrants		N/A	N/A	N/A	108,776^∝^	N/A	N/A	N/A	N/A	N/A	[45]
Italy	Administr-ative databases analysis	276 patients on RRT		N/A	58,218	N/A	48,835	N/A	N/A	N/A	N/A	N/A	[46]
Italy	Cohort study	1067 CKD patients		N/A	N/A	N/A	N/A	N/A	55,341± 17,646	40,460± 12,469	41,204± 11,013	39,709± 14,020	[31,47]
US	Medicare data analysis	Patients on different RRT ^Ω^		34,343	N/A	N/A	N/A	N/A	92,364	77,582	N/A	N/A	[48]
US	Medicare data analysis	Patients on different RRT ^Ω^		34,974	N/A	N/A	N/A	N/A	91,477	76,601	N/A	N/A	[49]
Sweden	Cohort study	3120 KTx recipients		N/A	76,293	15,564	N/A	N/A	N/A	N/A	N/A	N/A	[50]

^**&**^ Most studies estimated total health care costs based on primary care, in-patient, out-patient, diagnostics and medications/pharmaceuticals with exception of the following;

^+^ Studies estimated health care costs based on in-patient costs.

^%^ Studies estimated health care costs based on in-patient and out-patient costs

Abbreviations: SD: standard deviation; CI: confidence interval; CKD: chronic kidney disease; ADPKD: autosomal dominant polycystic kidney disease; ESRD: end-stage renal disease; RRT: renal replacement therapy; KTx: kidney transplantation; HD: haemodialysis; HHD: home haemodialysis; ICHD: in-center haemodialysis; PD: peritoneal dialysis; APD: automated peritoneal dialysis; CAPD: continuous ambulatory peritoneal dialysis; SHARP: The Study of Heart and Renal Protection; FNHIF: The French National Health Insurance funds; UKRR: UK Renal Registry data; N/A: non-available.

^@^ Combined KTx costs for those in the first or subsequent years.

^#^ Costs in patients with ADPKD.

^Φ^ Excluding the costs of maintenance dialysis and transplant surgery.

^δ^ Costs in patients with diabetic kidney disease.

^∝^ Costs were extrapolated from biannual or monthly costs.

^Ω^ Number of patients is non-available.

^¥^ Type of study that measures outcomes before and after the implementation of an intervention.

In general, patients receiving haemodialysis (HD) incurred higher costs compared to patients managed by PD. Concerning place of dialysis delivery, HHD costs were 49–65% of ICHD costs [38,40,44]. Furthermore, 11 studies estimated the annual health care costs of KTx in the range of $14,067 to $80,876 (Table 3). Studies showed that costs in the first year after KTx were substantially higher than in subsequent years, where they decreased dramatically. The costs of KTx were 21–88% of dialysis costs (Table 3). From a societal perspective, Eriksson et al. showed that costs incurred by patients who underwent KTx were 34–43% of costs incurred by dialysis patients (Table 2). Finally, Phair et al. conducted a cohort study specifically related to conservative (non-RRT) care for ESRD patients in England and Northern Ireland. They estimated the per patient mean annual health care costs to be US $7,411 (95% CI: 351–14,471) for patients receiving this care modality [8].

### 3.2. HRQoL associated with CKD

Thirty-six studies investigating the HRQoL associated with CKD and different treatment modalities were included. These studies used different HRQoL assessment tools, which comprised the EuroQol-5D (EQ-5D) [eight studies], the EuroQol visual analogue scale (EQ-VAS) [three studies], the Kidney Disease Quality of Life Short Form (KDQOL-SF^™^) [30 studies], and the Health Utilities Index (HUI) [one study].

#### 3.2.1. The EQ-5D and EQ-VAS instruments

The EQ-5D tool is a validated generic measure used to assess five dimensions of health (mobility, self-care, usual activities, pain and anxiety/depression), which can be converted into a single index score reflective of societal preferences [51]. The EQ-VAS is used for rating patients’ health using a scale ranging from 0 (worst health), to 100 (best health) [51]. The EQ-5D index scores were in the range of 0.80–0.86 for CKD stages 1–3 and decreased to 0.73–0.79 for CKD stages 4–5 (Table 4).

**Table 4:** Mean EQ-5D and VAS scores across different CKD stages and treatment modalities (Mean ± SD / (95% CI)).

Country	Participants	Tool	CKD 1/2	CKD 3	CKD 4	CKD 5	HD	PD	KTx	Reference
UK	512 KTx recipients	**EQ-5D-5L**	N/A	N/A	N/A	N/A	N/A	N/A	0.83	[52]
		**VAS**	N/A	N/A	N/A	N/A	N/A	N/A	N/A	
Nordics	243 patients	**EQ-5D-3L**	0.86 ± 0.16^#^		0.79 ± 0.23^#^		0.68 ± 0.30^#^		0.82± 0.21^#^	[53]
		**VAS**	81.7 ± 16.6^#^		70.4 ± 22.7^#^		60.1 ± 20.8^#^		76.7± 16.9^#^	
Europe	1336 CKD patients	**EQ-5D-3L**	N/A	0.76		N/A	N/A	N/A	N/A	[54]
		**VAS**	N/A	N/A		N/A	N/A	N/A	N/A	
Turkey	60 patients on RRT	**EQ-5D-3L**	N/A	N/A	N/A	N/A	0.60± 0.29	0.68± 0.33	N/A	[55]
		**VAS**	N/A	N/A	N/A	N/A	66.7± 22.3	58.1± 13.1	N/A	
France, Germany, Italy, UK, Spain, US	2233 CKD patients	**EQ-5D-3L**	N/A	0.82	0.78	0.71	N/A	N/A	N/A	[56]
		**VAS**	N/A	N/A	N/A	N/A	N/A	N/A	N/A	
UK	745 CKD patients	**EQ-5D-3L**	0.85 (0.70–1)	0.80 (0.68–1)	0.74 (0.62–0.85)	0.73 (0.62–1)	N/A	N/A	N/A	[57]
		**VAS**	50 (75–82)	70 (50–80)	60 (50–80)	55 (50–80)	N/A	N/A	N/A	
England & Ireland	893 patients on HD	**EQ-5D-3L**	N/A	N/A	N/A	N/A	0.58± 0.33	N/A	N/A	[58]
		**VAS**	N/A	N/A	N/A	N/A	58.3± 23.9	N/A	N/A	
Australia	95 CKD patients	**EQ-5D-3L**	N/A	N/A	N/A	0.75± 0.20	N/A	N/A	N/A	[59]
		**VAS**	N/A	N/A	N/A	N/A	N/A	N/A	N/A	

Cells are merged for studies which estimated combined score for stages 1–3, 3–4, and 4–5, as well as HD & PD. Abbreviations: SD: standard deviation; CI: confidence interval; CKD: chronic kidney disease; HD: haemodialysis; PD: peritoneal dialysis; KTx: kidney transplantation; EQ-5D: Euroqol-5D; VAS: visual analogue scale; N/A: non-available

^#^ Scores in patients with ADPKD.

Similarly, CKD stages 4–5 were associated with lower VAS scores compared to CKD stages 1–3 (Table 4). For RRT modalities, patients receiving dialysis had lower EQ-5D scores (0.58–0.68) than patients with a kidney transplant (0.82–0.83) (Table 4).

#### 3.2.2. The KDQOL-SF^™^ tool

The KDQOL-SF is a self-report measure consisting of generic and kidney-specific parts [60]. The generic part comprises eight domains that can be summarized in two summary scores; a physical component score (PCS) and a mental component score (MCS), whereas the specific part contains 12 domains addressing particular issues for CKD patients.

Patients with CKD stages 4–5 had the lowest mean PCS and MCS at 34.8–49.2 and 50.3–60.1 respectively [53,61–67] (S2 Table). For RRT treatments, KTx recipients reported higher mean generic and specific scores relative to those on dialysis [68–83] (S2–S4 Tables). Nagaraja et al. [44] converted the SF-36 data into SF-6D utility scores and showed a similar relationship; kidney transplantation was associated with better mean SF-6D utility scores relative to HD (0.65± SD: 0.13 *vs* 0.52± SD: 0.11, respectively).

Peritoneal dialysis was associated with better scores than HD for both generic and kidney specific domains [84,85]. Nagaraja et al. [44], on the other hand, reported that mean SF-6D utility scores among patients managed by PD were similar to those receiving HD (0.53 ± 0.10 *vs* 0.52 ± 0.11, respectively). Regarding place of dialysis delivery, Nagaraja et al. [44] reported no significant difference between mean SF-6D utility scores of HHD and ICHD (0.52 ± 0.15 *vs* 0.52 ± 0.11, respectively).

Finally, Shah et al. [86,87] indicated that ESRD elderly patients managed with conservative care exhibited significantly higher mean SF-6D utility scores compared to those receiving dialysis (0.65 ± 0.15, *vs* 0.61 ± 0.13, respectively).

#### 3.2.3. The HUI system

The HUI system is a generic, preference-based measure used to produce utility scores providing a summary index of HRQoL on a (0.0–1.0) scale [88]. A cross-sectional study by Garg et al. [76] reported that HHD was associated with better mean scores among ESRD patients, relative to ICHD (0.64 ± 0.34 *vs* 0.53 ± 0.38, respectively).

### 3.3. LE among CKD patients

In total, seven studies addressing LE and survival among CKD patients were included.

Bowling et al. [89] conducted a cohort study in 357,632 CKD patients aged ≥ 70 years and showed that median survival time decreased with progression in either age or CKD stage; patients aged ≥ 85 years had the lowest survival ranging from 1.9 (CKD stage 4) to 3.2 years (CKD stage 2). Tamura et al. [90] estimated survival among 73,349 older ESRD patients (≥ 60 years) initiating dialysis at different kidney function levels. The study revealed that median survival was diminished upon initiating dialysis at higher GFRs as well as with older age. For example, patients aged ≥ 85 years exhibited the lowest survival from initiating dialysis varying from 1.8 years at an eGFR of 9–12 ml/min/1.73 m^2^) to 2.0 years at an eGFR < 6 ml/min/1.73 m^2^.

Steenkamp et al. [91] analyzed the 2014–2015 UK renal registry data of 7,626 ESRD patients, and showed that survival probabilities (adjusted to age 60) for 15 months after starting RRT (dialysis or KTx) was 90.1%. On the other hand, Couchoud et al. [92] investigated restricted mean survival time amongst 67,258 ESRD patients according to their age, gender, and diabetes status for the first 15 years following RRT initiation. The study demonstrated that both males and females aged 18 with diabetes experienced lower survival time (12.7 years for either gender) compared to those without diabetes (males; 14.3 years and females; 14.2 years). In contrast, survival time in both diabetic and non-diabetic patients aged 90 years was nearly similar (males: 1.6 vs. 1.8 years, respectively and females: 1.9 vs. 2 years, respectively).

Two reports by the US Renal Data System (USRDS) [93,94] compared survival probabilities for ESRD patients on HD and PD over the period (2000–2009), and showed that HD was consistently associated with lower survival than PD. The same reports by the USRDS also investigated the difference in survival between different KTx types over the same period, and demonstrated that living donor transplant recipients always showed higher survival, relative to deceased donor kidney transplant recipients. The 2016 USRDS report [94] showed that KTx was associated with higher LE (7.7 for males and 8.7 for females) than dialysis (3.2 for males and 3.5 for females) in the age group 75–79 years, with similar results shown across all age groups.

Finally, a cohort study by Verberne et al. [95] compared survival time among 311 ESRD patients (age ≥ 70 years) receiving either RRT (dialysis or KTx) or conservative care and reported survival of 3.1 year and 1.5 years, respectively.

### 3.4. Consultation exercise

All nephrologists concurred with the review findings in relation to the increase in costs and decrease in HRQoL as CKD stage advanced. The nephrologists suggested that costs at different stages could vary considerably depending on what is bundled into the episode of care (e.g. hospital clinic or admission costs, and training of staff), aetiology of CKD and associated comorbidities. These factors may help explain the heterogeneity with respect to costs found in this study. The nephrologists agreed that societal costs might be inaccurately estimated and poorly captured in the current reports. Examples of societal costs include productivity losses (valued, for example, through the human capital approach) amongst CKD patients, donors and/or carers due to either absenteeism (work absences) or presenteeism (reduced productivity among those with CKD in work). Societal costs also include transportation costs, and avoidable co-morbidity such as depression occasioned while on HD and awaiting transplantation.

The nephrologists attributed the higher costs in the US, relative to Europe, to systems that carefully itemize more costs. In the US, in contrast to Europe, there may be more comprehensive attribution of health care expenditure resulting in higher unit costs (i.e. prices for drugs and procedures), higher rates of comorbidities in CKD/ESRD patients (those presenting may have been sicker), and a lower threshold for investigation in some clinical settings (whether as a result of a higher likelihood to practice defensive medicine, an ease of access to diagnostic investigations or an ability to recoup costs from insurers). They suggested that HRQoL data should be interpreted cautiously due to potential confounders (e.g. comorbidities and higher prevalence of depression amongst CKD patients).

Concerning different RRT modalities, all nephrologists confirmed that KTx is accepted to be consistently associated with lower costs, better HRQoL and longer survival compared to dialysis. They also supported the review findings regarding the substantial decline in costs in subsequent years following the first year after KTx, and attributed that to reduced burden of procedures, hospitalizations and outpatient appointments following stabilization of post-transplant follow-up and to the availability of relatively cheap generic immunosuppressive drugs. The nephrologists suggested that HRQoL and LE data for KTx might be subject to a degree of selection bias, as healthier patients are more likely to be transplanted.

All nephrologists acknowledged that the review findings would be useful for commissioners considering the cost-effectiveness of new therapies. They also advised that the study would inform future research, resource allocation, training of health personnel, preventative health care, and opportunities for reducing costs (e.g. telehealth, standardisation of medication use, cost negotiations with pharma).

## 4. Discussion

This scoping review has explored the reported burden associated with CKD in order to inform provision of convenient renal services and economic evaluations of innovative therapies. While broadly consistent with the assumptions and experiences of practicing nephrologists, the findings of this review provide a deeper quantitative understanding of the influence of CKD on costs, HRQoL and LE in developed countries during the last five years.

The results showed great variations in costs across different CKD stages, where costs substantially increased with each advance in CKD stage. Most studies (86%) estimated total health care costs among CKD patients. From the health system perspective, the progression from CKD stages 1–2 to CKD stages 3a-3b was associated with a 1.1–1.7-fold increase in per patient mean annual costs, with CKD stages 1–2 demonstrating the lowest costs ($1,768-$20,104), with Europe and the US at the lower and the higher limits of the range, respectively. The transition from CKD stage 3 to CKD stages 4–5 was associated with a 1.3–4.2-fold rise in costs, with ESRD showing the highest costs ranging from $20,110 to $100,593, with Europe and the US at the lower and the higher limits of the range, respectively. Although health care costs of CKD show some variation, they are consistent in the sense that the US and Europe have the highest and lowest costs for all stages, respectively. The substantial variation between Europe and the US may be linked to differences in health care accountancy, difference in clinical practices (e.g. different threshold for investigation in some clinical places) and/or differences in the cost of care between countries [96]. While costs vary between countries in a manner that would require further research to explain, the increase in costs with advanced CKD stages is consistent within country analyses.

The review produced limited data addressing costs from a societal perspective in Europe and none in the US. These costs were also highest amongst patients with CKD stages 4–5 ($10,750- $28,428). Productivity loss was highest in the Nordic countries, accounting for 2/3 of the total societal costs among patients with CKD stages 4–5 [19]. Despite the potential time commitment involved in dialysis and/or in transplantation, there has been limited evidence with respect to estimation of productivity losses associated with RRT. Similarly, while previous research has documented the relationship between dialysis and depression [97], and the potential impact of depression on productivity [98], there is also a gap in research examining the impact of comorbidities on the productivity of ESRD patients.

Different HRQoL assessment tools showed variations in HRQoL scores across CKD stages, where scores showed a notable decrease with each progressive CKD stage. The transition from CKD stages 1–3 to CKD stages 4–5 was associated with a decrease of 8–11% in mean EQ-5D index scores, with CKD stages 4–5 showing the lowest scores ranging from 0.74 to 0.79. A systematic literature review of 21 studies from developed countries showed that mean EQ-5D scores among patients with type 2 diabetes (without complications) were in the range of 0.71–0.94 [99]. A systematic review of 60 studies from westernized societies showed that mean EQ-5D score was even lower for the late heart failure stages (0.51) [100]. These low scores revealed the poor outcomes associated with these interrelated chronic diseases, highlighting the need for effective preventive measures to slow their progression.

This study produced limited LE data among CKD patients, where available this suggested that LE substantially decreased with each age group and with worse kidney function. Similar findings have also been reported by Turin et al. in a large abridged life table analysis study in Canada [101]. The low survival among older patients starting dialysis at higher eGFR levels is likely confounded by the indications to start dialysis (i.e.co-morbidities such as heart failure or respiratory disease).

Our study explored costs and outcomes associated with different CKD treatment modalities. From a health system perspective, the costs of HD were 1.1–1.8-fold those of PD. This has also previously been reported in a cohort study by Berger et al. [7]. Regarding place of dialysis delivery, costs of ICHD were 1.5-2-fold those of HHD. The study results highlighted the substantial variations in transplantation costs, which were estimated to be in the range of $14,067 to $31,115, and $34,343 to $80,876, from a health system perspective, in Europe and the US, respectively. This considerable variation may be linked to difference in health care accountancy, discrepancy in practices between countries or simply differences in the cost of care between countries. Our study demonstrated that costs of dialysis were 1.1-6-fold and 2.3–2.9-fold those of KTx, from a health system and societal perspective respectively. Among all treatment modalities, KTx exhibited the highest mean EQ-5D scores, ranging from 0.82 to 0.83. Physical and mental component scores were also highest among patients with KTx, and were reported to be in the range of 42–47 and 43–54, respectively. This has also been reported in a systematic review of 110 studies by Tonelli et al. [6]. Our study also showed that KTx was associated with higher LE among both women and men across all age groups compared to dialysis modality though the potential for selection bias effects here must be borne in mind. Not unsurprisingly, conservative care had the lowest health care costs ($7,411) as shown by Phair et al. [8], though these results should be treated with some caution given the sample size (the number of participants who completed this study (n = 8)).

## 5. Strengths and limitations

This review adopted a comprehensive systematic search strategy for scoping literature to address its objectives. A broad range of study designs/methodologies from both recent peer- reviewed and non-peer reviewed literature were included. Furthermore, costs in this study were adjusted for inflation and purchasing power parity to improve comparability. This study also considered a consultation exercise with nephrologists. The review nevertheless still had some limitations. The authors only assessed and included English language articles, which posed a possibility of missing potential papers from continental Europe. The quality of the included studies was not assessed in order to include an extensive range of evidence from both peer-reviewed and grey literature and provide a better understanding of the burden associated with CKD. This review included only studies from North America, Europe, and Australia, which may not make the study findings transferable to developing countries, where the delivery of health care services is different and RRT is not available for many ESRD patients. Since, most studies examined total healthcare costs, irrespective of whether it is related or not related to CKD, the itemisation of all services and costs was infeasible.

## 6. Conclusions and implications

Costs and outcomes associated with CKD varied considerably across different stages. From a health system perspective, the transition from CKD stage 3 to CKD stages 4–5 was associated with a 1.3–4.2-fold increase in costs, with ESRD showing the highest costs ($20,110-$100,593). The progression from CKD stages 1–3 to CKD stages 4–5 was associated with a decline of 8–11% in mean EQ-5D index scores, with CKD stages 4–5 demonstrating the lowest scores (0.74–0.79). Recent studies of LE associated with different CKD stages in different countries have been limited in number and scope. Amongst treatment modalities, KTx showed the lowest costs ($14,067-$80,876), highest EQ-5D scores (0.82–0.83), and longest LE among both males and females across all age groups.

In addition to identifying gaps in knowledge, the current study contributes to a better understanding of the economic costs likely to attend increases in CKD prevalence, informing health care planning and resourcing in developed countries. This study can also inform the development of cost-effectiveness models of novel therapies that prevent or slow the progression of CKD. Based on the study findings, the following areas are recommended for future research;

- Investigation of CKD aetiology-specific costs and HRQoL as they could substantially vary depending on aetiology of CKD.
- A more comprehensive and consistent estimation of societal costs associated with different CKD stages and RRT modalities with particular emphasis on productivity losses and costs related to induced conditions such as depression.
- A careful itemization of healthcare costs aspects and examining whether they are paid for by a third part or out of pocket.
- Conducting abridged life table analyses for estimation of LE across different CKD stages in different countries.

## Supporting information

S1 Appendix PRISMA checklist. (DOCX)

S2 Appendix The key concepts and final selected search terms of the search strategy. (DOCX)

S3 Appendix Consultation exercise survey. (DOCX)

S1 Table Numerical distribution of the included studies. (DOCX)

S2 Table PCS and MCS across different CKD stages and treatment modalities. (DOCX)

S3 Table Generic domains scores associated with ESRD and different treatment modalities. (DOCX)

S4 Table Kidney-specific domains scores associated with ESRD and different treatment modalities. (DOCX)
