# Supplementary material for: The impact of chronic kidney disease on developed countries from a health economics perspective: A systematic scoping review
Source: PLoS One. 2020 Mar 24;15(3):e0230512. doi: 10.1371/journal.pone.0230512 (PMC7092970; doi:10.1371/journal.pone.0230512)
Supplement: S2 Appendix — (DOCX) [file pone.0230512.s002.docx]

**S2 Appendix. The key concepts and final selected search terms of the search strategy**

| Concept 1 | Concept 2 | Concept 3 | Concept 4 |
| --- | --- | --- | --- |
| (Societal cost) | (HRQoL) | (LE) | (CKD) |
| **“Societal cost*” OR**  **“healthcare cost*” OR**  **employment*** **OR**  **absenteeism* OR**  **“productivity loss” OR**  **productivit*** | **“Health related quality of life*” OR hql***  **hqol* OR euroqol* OR eq5d* OR eq5* OR SF36* OR “short form 36*” OR SF12* OR**  **“short form 12*” OR “health utility*” OR “utility score*” OR “utility value*” OR hui** | **“life expectanc*” OR**  **survival* OR**  **“average life” OR “average liv*”** | **“chronic kidney disease*” OR ESRD OR “end stage renal disease*” OR**  **“chronic renal disease*” OR “chronic renal insufficienc*” OR DKD OR “diabetic kidney disease*” OR**  **“renal replacement therap*” OR RRT OR “kidney transplant*” OR “dialys*”** |
