# Supplementary material for: The impact of chronic kidney disease on developed countries from a health economics perspective: A systematic scoping review
Source: PLoS One. 2020 Mar 24;15(3):e0230512. doi: 10.1371/journal.pone.0230512 (PMC7092970; doi:10.1371/journal.pone.0230512)
Supplement: S3 Appendix — (DOCX) [file pone.0230512.s003.docx]

**S3 Appendix. Consultation exercise survey**

| **Kidney Study Consultation**  *Thank you for accepting to participate in this important survey exploring your opinion on the findings of our study investigating costs and outcomes associated with chronic kidney disease. This survey takes 10-15 minutes to complete. Your answers will help validate our study findings and inform developing sensible recommendations for future research.* |
| --- |
| **1.** Does this study reflect your experience with how costs might increase across CKD stages? |
| **2.** Does this study reflect your experience with how HRQoL might decrease across CKD stages? |
| **3.** Do the study findings reflect your experience in terms of how transplantation is associated with lower costs, and higher HRQoL and life expectancy compared to dialysis? |
| **4.** Do you think the differences in costs between the US and different European countries (e.g. UK, Italy,..etc) might best be explained by:   - 1. Differences in practices/guidelines?   2. Differences in what is included in a given cost – e.g. more diagnostics tests   3. Differences in unit costs – e.g. higher staff pay in the USA   4. All of the above |
| **5.** Do you think the study has omitted any relevant aspect of CKD burden? For example, do you think there may be aspects of cost that may not be/are not captured in those cited – e.g. transport related to kidneys being transported; impact on carers/partners in terms of health or lost productivity? |
| **6**. Do you think the study findings are transferable i.e. useful in terms of giving healthcare providers some insight into the impact of CKD on individuals, societies and health systems in developed countries? |
| **7.** From your perspective, what are the possible implications of this study? |
