# Supplementary material for: The impact of chronic kidney disease on developed countries from a health economics perspective: A systematic scoping review
Source: PLoS One. 2020 Mar 24;15(3):e0230512. doi: 10.1371/journal.pone.0230512 (PMC7092970; doi:10.1371/journal.pone.0230512)
Supplement: S1 Table — (DOCX) [file pone.0230512.s004.docx]

**S1 Table. Numerical distribution of the included studies**

| **Study category** | **Number of studies n (%)** |
| --- | --- |
| **Country of the study** | |
| Europe | 48 (62%) |
| North America (US & Canada) | 24 (31%) |
| Australia | 6 (7%) |
| **Study type** | |
| Journal article | 52 (67%) |
| Conference proceeding | 18 (23%) |
| Report | 8 (10%) |
| **Study design** | |
| Cross-sectional study | 30 (38%) |
| Cohort study | 26 (33%) |
| Secondary analysis | 17 (22%) |
| Case-control study | 2 (3%) |
| Clinical trial | 2 (3%) |
| Before-after study design | 1 (1%) |
