# Supplementary material for: The impact of chronic kidney disease on developed countries from a health economics perspective: A systematic scoping review
Source: PLoS One. 2020 Mar 24;15(3):e0230512. doi: 10.1371/journal.pone.0230512 (PMC7092970; doi:10.1371/journal.pone.0230512)
Supplement: S2 Table — (DOCX) [file pone.0230512.s005.docx]

**S2** **Table. PCS and MCS across different CKD stages and treatment modalities (Mean ± SD / (95% CI))**

| **Country** | **Study subjects** | **Tool, Domain** | **CKD 1/2** | **CKD 3** | **CKD 4** | **CKD 5** | **HHD** | **ICHD** | **HD and/or PD** | **KTx** | **Reference** |
| --- | --- | --- | --- | --- | --- | --- | --- | --- | --- | --- | --- |
| France | 29689 CKD patients | SF-12, PCS | N/A | 42.7 | 40.4 | N/A | N/A | N/A | 35.8 | 45.8 | [61] |
|  |  | SF-12, MCS | N/A | 47.9 | 47.5 | N/A | N/A | N/A | 40.7 | 46.0 |  |
| Poland | 118 KTx recipients | SF-36, PCS | N/A | N/A | N/A | N/A | N/A | N/A | N/A | 42.2±8.4 | [68] |
|  |  | SF-36, MCS | N/A | N/A | N/A | N/A | N/A | N/A | N/A | 43.1±10.1 |  |
| Canada | 33 patients on RRT | SF-36, PCS | N/A | N/A | N/A | N/A | 37.3 | 34.0 | N/A | N/A | [69] |
|  |  | SF-36, MCS | N/A | N/A | N/A | N/A | 28.3 | 53.0 | N/A | N/A |  |
| UK | 74 patients with CKD 5 | SF-36, PCS | N/A | N/A | N/A | 44.6± 5.8 | N/A | N/A | N/A | N/A | [62] |
|  |  | SF-36, MCS | N/A | N/A | N/A | 48.1± 8.8 | N/A | N/A | N/A | N/A |  |
| Nordic countries | 243 ADPKD patients | SF-12, PCS | 51.2 ± 7.5^#^ | | 43.0 ± 10.3^#^ | | N/A | N/A | 34.9± 11.1^#^ | 45.0± 11.1^#^ | [53] |
|  |  | SF-12, MCS | 50.5 ± 9.6^#^ | | 52.7 ± 8.4^#^ | | N/A | N/A | 47.0± 9.0^#^ | 52.7± 9.0^#^ |  |
| Canada & US | 332 patients on RRT | SF-36, PCS | N/A | N/A | N/A | N/A | 38.2± 8.3 | 38.0± 9.7 | N/A | N/A | [76] |
|  |  | SF-36, MCS | N/A | N/A | N/A | N/A | 45.9± 12.6 | 46.0± 10.3 | N/A | N/A |  |
| The Netherlands | 72 patients on RRT | SF-36, PCS | N/A | N/A | N/A | N/A | 43.0± 8.0 | N/A | N/A | 47± 10 | [77] |
|  |  | SF-36, MCS | N/A | N/A | N/A | N/A | 52.0± 11.0 | N/A | N/A | 51± 10 |  |
| Denmark | 81 patients on HD | SF-36, PCS | N/A | N/A | N/A | N/A | N/A | 37.4± 10.2 | N/A | N/A | [78] |
|  |  | SF-36, MCS | N/A | N/A | N/A | N/A | N/A | 49.1± 11.9 | N/A | N/A |  |
| Norway | 261 elderly patients with CKD 5 | SF-36, PCS | N/A | N/A | N/A | 38.1 (36.6 -39.7) | N/A | N/A | N/A | N/A | [63] |
|  |  | SF-36, MCS | N/A | N/A | N/A | 44.7 (43.0 -46.4) | N/A | N/A | N/A | N/A |  |
| The Netherlands | 1379 patients on ICHD | SF-36, PCS | N/A | N/A | N/A | N/A | N/A | 38.4 (37.9 -38.9) | N/A | N/A | [79] |
|  |  | SF-36, MCS | N/A | N/A | N/A | N/A | N/A | 44.8 (44.3 - 45.5) | N/A | N/A |  |
|  |  | SF-12, PCS | N/A | N/A | N/A | N/A | N/A | 37.8 (37.3 - 38.3) | N/A | N/A |  |
|  |  | SF-12, MCS | N/A | N/A | N/A | N/A | N/A | 46.4 (45.9 - 47.0) | N/A | N/A |  |
| Spain | 52 patients on HD | SF-36, PCS | N/A | N/A | N/A | N/A | N/A | 53.7± 22.8 | N/A | N/A | [80] |
|  |  | SF-36, MCS | N/A | N/A | N/A | N/A | N/A | 63.6± 25.2 | N/A | N/A |  |
| France | 1424 KTx patients | SF-36, PCS | N/A | N/A | N/A | N/A | N/A | N/A | N/A | 45.8± 9.7 | [81] |
|  |  | SF-36, MCS | N/A | N/A | N/A | N/A | N/A | N/A | N/A | 46.0± 10.5 |  |
| Portugal | 322 patients on HD | SF-36, PCS | N/A | N/A | N/A | N/A | N/A | 49.8± 9.4 | N/A | N/A | [82] |
|  |  | SF-36, MCS | N/A | N/A | N/A | N/A | N/A | 50.2± 9.5 | N/A | N/A |  |
| UK | 497 patients on RRT | SF-36, PCS | N/A | N/A | N/A | N/A | 31.6± 8.3 | 32.4± 9.7 | N/A | 43.5± 11.1 | [75] |
|  |  | SF-36, MCS | N/A | N/A | N/A | N/A | 41.6± 12.6 | 42.5± 11.8 | N/A | 46.8± 10.1 |  |
| Ireland | 82 DKD patients | SF-36, PCS | 31.9^φ^ | | | | N/A | 29.2^φ^ | N/A | N/A | [83] |
|  |  | SF-36, MCS | 53.39^φ^ | | | | N/A | 60.4^φ^ | N/A | N/A |  |
| US | 3,409 CKD patients | SF-12, PCS | N/A | N/A | 46.8± 9.9 | N/A | N/A | N/A | N/A | N/A | [64] |
|  |  | SF-12, MCS | N/A | N/A | 51.1± 9.6 | N/A | N/A | N/A | N/A | N/A |  |
| US | 3837 patients with CKD | SF-36, PCS | N/A | 39.9± 11.2 | 37.1± 10.7 | N/A | N/A | N/A | N/A | N/A | [65] |
|  |  | SF-36, MCS | N/A | 50.3± 10.4 | 49.5± 11.1 | N/A | N/A | N/A | N/A | N/A |  |
| UK | 139 CKD Patients | SF-36, PCS | 75.3± 21.5 | 62.6± 24.5 | 49.2± 24.2 | | N/A | N/A | N/A | N/A | [66] |
|  |  | SF-36, MCS | 69.6± 22.1 | 65.9± 19.2 | 60.1± 24.0 | | N/A | N/A | N/A | N/A |  |
| Germany, Italy, UK Poland, Sweden & Netherlands | 1079 CKD 4/5 patients aged ≥65 | SF-36, PCS | N/A | N/A | 34.8± 12.1 | | N/A | N/A | N/A | N/A | [67] |
|  |  | SF-36, MCS | N/A | N/A | 50.3± 10.9 | | N/A | N/A | N/A | N/A |  |
| US | 213 KTx recipients | SF-36, PCS | N/A | N/A | N/A | N/A | N/A | N/A | N/A | 41.6± 10.1 | [70] |
|  |  | SF-36, MCS | N/A | N/A | N/A | N/A | N/A | N/A | N/A | 53.5± 8.4 |  |
| Australia | 308 patients with DKD | SF-36, PCS | N/A | 35.7± 11.3^φ^ | | | N/A | N/A | 33.0± 10.3^φ^ | N/A | [71,72] |
|  |  | SF-36, MCS | N/A | 47.2± 11.1^φ^ | | | N/A | N/A | 46.4± 10.1^φ^ | N/A |  |

Cells are merged for studies which estimated combined score for stages 1-3, 3-5, 4-5, and 1-5. Abbreviations: PCS= physical component score; MCS= mental component score; CKD= chromic kidney disease; SD= standard deviation; CI= confidence interval; HHD= home haemodialysis; ICHD= in-center haemodialysis; KTx= kidney transplantation; ADPKD= autosomal dominant polycystic kidney disease; DKD= diabetic kidney disease; N/A= non-available. ^#^ Scores in patients with ADPKD. ^φ^ Scores in DKD patients
