# Supplementary material for: The impact of chronic kidney disease on developed countries from a health economics perspective: A systematic scoping review
Source: PLoS One. 2020 Mar 24;15(3):e0230512. doi: 10.1371/journal.pone.0230512 (PMC7092970; doi:10.1371/journal.pone.0230512)
Supplement: S3 Table — (DOCX) [file pone.0230512.s006.docx]

**S3 Table. Generic domains scores associated with ESRD and different treatment modalities (Mean ± SD / (95% CI))**

| **Country** | **Stage/ RRT** | **Physical Function** | **Social Function** | **Role Physical** | **Role Emotional** | **Mental Health** | **Vitality** | **Bodily Pain** | **General Health** | **Reference** |
| --- | --- | --- | --- | --- | --- | --- | --- | --- | --- | --- |
| Poland | ESRD | N/A | N/A | N/A | N/A | N/A | N/A | N/A | N/A | [68] |
|  | HD | N/A | N/A | N/A | N/A | N/A | N/A | N/A | N/A |  |
|  | PD | N/A | N/A | N/A | N/A | N/A | N/A | N/A | N/A |  |
|  | KTx | 73.3± 24.2 | 66.1± 26.9 | 23.7± 36.5 | 37.3± 43.6 | 58.6± 17.8 | 54.7± 17.7 | 66.2± 27.4 | 54.2± 23.4 |  |
| UK | ESRD | 44.0± 6.4 | 46.2± 8.7 | 44.6± 6.0 | 49.8± 8.9 | 48.7± 9.8 | 41.5± 8.7 | 53.6± 9.7 | 41.2± 8.1 | [62] |
|  | HD | N/A | N/A | N/A | N/A | N/A | N/A | N/A | N/A |  |
|  | PD | N/A | N/A | N/A | N/A | N/A | N/A | N/A | N/A |  |
|  | KTx | N/A | N/A | N/A | N/A | N/A | N/A | N/A | N/A |  |
| Denmark | ESRD | N/A | N/A | N/A | N/A | N/A | N/A | N/A | N/A | [78] |
|  | HD | 58.7± 26.0 | 73.8± 27.7 | 33.7± 37.9 | 55.2± 40.9 | 77.0± 22.4 | 48.3± 26.5 | 63.6± 31.8 | 44.2± 22.4 |  |
|  | PD | N/A | N/A | N/A | N/A | N/A | N/A | N/A | N/A |  |
|  | KTx | N/A | N/A | N/A | N/A | N/A | N/A | N/A | N/A |  |
| Poland | ESRD | N/A | N/A | N/A | N/A | N/A | N/A | N/A | N/A | [84] |
|  | HD | 58± 25 | 49± 21 | 15± 31 | 30± 39 | 45± 18 | 41± 18 | 52± 27 | 34± 19 |  |
|  | PD | 55± 25 | 52± 30 | 23± 35 | 45± 46 | 44± 22 | 43± 23 | 56± 28 | 32± 15 |  |
|  | KTx | 74± 25 | 54± 17 | 52± 44 | 65± 41 | 63± 19 | 60± 17 | 67± 29 | 40± 14 |  |
| Norway | ESRD | N/A | N/A | N/A | N/A | N/A | N/A | N/A | N/A | [73] |
|  | HD | 68± 24 | 70± 29 | 36± 41 | 65± 41 | 77± 18 | 46± 22 | 66± 27 | 47± 22 |  |
|  | PD |  |  |  |  |  |  |  |  |  |
|  | KTx | 74± 28 | 81± 26 | 54± 44 | 71± 42 | 78± 19 | 55± 24 | 73± 28 | 59± 26 |  |
| Norway | ESRD | N/A | N/A | N/A | N/A | N/A | N/A | N/A | N/A | [74] |
|  | HD | 66 | 64 | 28 | 62 | 79 | 42 | 63 | 51 |  |
|  | PD |  |  |  |  |  |  |  |  |  |
|  | KTx | 73 | 67 | 39 | 66 | 84 | 61 | 75 | 70 |  |
| Norway | ESRD | 59.3 (55.6-63.0) | 51.9 (48.5-55.3) | 25.1 (18.9-31.2) | 54.5 (47.1-61.8) | 74.0 (71.0-77.0) | 40.5 (37.0 -43.9) | 60.5 (56.0-65.1) | 47.4 (43.6-51.1) | [63] |
|  | HD | N/A | N/A | N/A | N/A | N/A | N/A | N/A | N/A |  |
|  | PD | N/A | N/A | N/A | N/A | N/A | N/A | N/A | N/A |  |
|  | KTx | N/A | N/A | N/A | N/A | N/A | N/A | N/A | N/A |  |
| France | ESRD | N/A | N/A | N/A | N/A | N/A | N/A | N/A | N/A | [81] |
|  | HD | N/A | N/A | N/A | N/A | N/A | N/A | N/A | N/A |  |
|  | PD | N/A | N/A | N/A | N/A | N/A | N/A | N/A | N/A |  |
|  | KTx | 74.8± 24.3 | 74.9± 23.6 | 64.4± 41.3 | 68.8± 41.3 | 65.5± 18.7 | 53.3± 19.3 | 68.3± 25.8 | 55.4± 21 |  |
| Portugal | ESRD | N/A | N/A | N/A | N/A | N/A | N/A | N/A | N/A | [82] |
|  | HD | 44.4± 30.8 | 68.2± 34.5 | 26.9± 32.7 | 45.3± 19.8 | 61.5± 24.9 | 48.3± 19.8 | 63.1± 29.8 | 35.1± 21.9 |  |
|  | PD | N/A | N/A | N/A | N/A | N/A | N/A | N/A | N/A |  |
|  | KTx | N/A | N/A | N/A | N/A | N/A | N/A | N/A | N/A |  |
| Spain | ESRD | N/A | N/A | N/A | N/A | N/A | N/A | N/A | N/A | [85] |
|  | HD | 43.9± 29.2 | 52.4± 33.9 | 20.3± 35.5 | 54.3± 47.3 | 56.1± 25.7 | 39.8± 25.8 | 48.2± 31.1 | 38.1± 19.6 |  |
|  | PD | 53.3± 29.5 | 53.5± 31.2 | 22.7± 39.8 | 57.3± 49.5 | 55.2± 23.7 | 40.3± 24.8 | 49.1± 27.5 | 37.2± 17.9 |  |
|  | KTx | N/A | N/A | N/A | N/A | N/A | N/A | N/A | N/A |  |
| UK | ESRD | 51.2± 29.3 | 68.8± 27.6 | 38.9± 42.0 | 63.0± 42.0 | 72.8± 21.4 | 36.0± 24.4 | 67.7±27.9 | 41.0± 21.9 | [66] |
|  | HD | N/A | N/A | N/A | N/A | N/A | N/A | N/A | N/A |  |
|  | PD | N/A | N/A | N/A | N/A | N/A | N/A | N/A | N/A |  |
|  | KTx | N/A | N/A | N/A | N/A | N/A | N/A | N/A | N/A |  |

For each scale assessed, item scores were summed and transformed using a scale of zero-100, with higher scores showing better HRQoL. Abbreviations: ESRD= end-stage renal disease; SD= standard deviation; CI= confidence interval; RRT= renal replacement therapy; HD= haemodialysis; PD= peritoneal dialysis; KTx= kidney transplantation; N/A= non-available.
