# Supplementary material for: The impact of chronic kidney disease on developed countries from a health economics perspective: A systematic scoping review
Source: PLoS One. 2020 Mar 24;15(3):e0230512. doi: 10.1371/journal.pone.0230512 (PMC7092970; doi:10.1371/journal.pone.0230512)
Supplement: S4 Table — (DOCX) [file pone.0230512.s007.docx]

**S4 Table. Kidney-specific domains scores associated with ESRD and different treatment modalities (Mean ± SD / (95% CI))**

| **Country** | **Stage/ RRT** | **Symp** | **EKD** | **BKD** | **Work status** | **Cogn** | **QSI** | **Sex** | **Sleep** | **Soc** | **SE** | **PS** | **Overall health** | **Reference** |
| --- | --- | --- | --- | --- | --- | --- | --- | --- | --- | --- | --- | --- | --- | --- |
| Poland | ESRD | N/A | N/A | N/A | N/A | N/A | N/A | N/A | N/A | N/A | N/A | N/A | N/A | [68] |
|  | HD | N/A | N/A | N/A | N/A | N/A | N/A | N/A | N/A | N/A | N/A | N/A | N/A |  |
|  | PD | N/A | N/A | N/A | N/A | N/A | N/A | N/A | N/A | N/A | N/A | N/A | N/A |  |
|  | KTx | 80.1± 15.7 | 75.8± 21.6 | 60.8± 29.8 | 53.8± 45.4 | 68.5± 21.4 | 71.9± 19.2 | 70.9± 33.2 | 65.2± 22.0 | 72.0± 24.6 | 58.5± 34.4 | 49.3± 30.5 | 66.9± 20.0 |  |
| The  Netherlands | ESRD | N/A | N/A | N/A | N/A | N/A | N/A | N/A | N/A | N/A | N/A | N/A | N/A | [77] |
|  | HD | N/A | N/A | N/A | N/A | N/A | N/A | N/A | N/A | N/A | N/A | N/A | N/A |  |
|  | PD | N/A | N/A | N/A | N/A | N/A | N/A | N/A | N/A | N/A | N/A | N/A | N/A |  |
|  | KTx | 86 ±11 | 86 ±14 | 75 ±27 | N/A | 81± 19 | 79± 15 | 72± 30 | 66± 23 | 87± 21 | N/A | N/A | 70± 16 |  |
| Denmark | ESRD | N/A | N/A | N/A | N/A | N/A | N/A | N/A | N/A | N/A | N/A | N/A | N/A | [78] |
|  | HD | 78.7± 13.6 | 68.1± 19.9 | 39.7± 25.8 | 21.1± 32.9 | 85.0± 21.1 | 76.5± 18.1 | 77.5± 30.2 | 64.1± 21.9 | 82.0± 24.0 | 82.6± 23.0 | 82.7± 17.1 | 61.4± 21.9 |  |
|  | PD | N/A | N/A | N/A | N/A | N/A | N/A | N/A | N/A | N/A | N/A | N/A | N/A |  |
|  | KTx | N/A | N/A | N/A | N/A | N/A | N/A | N/A | N/A | N/A | N/A | N/A | N/A |  |
| Poland | ESRD | N/A | N/A | N/A | N/A | N/A | N/A | N/A | N/A | N/A | N/A | N/A | N/A | [84] |
|  | HD | 61± 18 | 52± 16 | 35± 20 | 27± 40 | 67± 24 | 72± 20 | 54± 31 | 51± 22 | 69± 18 | 79± 22 | 41± 19 | N/A |  |
|  | PD | 65± 20 | 56± 21 | 38± 27 | 34± 45 | 59± 25 | 69± 23 | 61± 30 | 51± 27 | 75± 25 | 84± 23 | 46± 24 | N/A |  |
|  | KTx | 85± 14 | 78± 20 | 39± 19 | 33± 41 | 77± 23 | 79± 19 | 72± 32 | 69± 22 | 83± 17 | 83± 19 | 57± 22 | N/A |  |
| Norway | ESRD | N/A | N/A | N/A | N/A | N/A | N/A | N/A | N/A | N/A | N/A | N/A | N/A | [73] |
|  | HD | 74± 16 | 69± 18 | 39± 26 | 21± 35 | 85± 17 | 82± 18 | 71± 31 | 61± 21 | 78± 28 | N/A | N/A | N/A |  |
|  | PD |  |  |  |  |  |  |  |  |  |  |  |  |  |
|  | KTx | 82± 15 | 84± 16 | 73± 27 | 45± 42 | 88± 14 | 80± 18 | 85± 20 | 69± 20 | 83± 27 | N/A | N/A | N/A |  |
| Norway | ESRD | 72.8 (70.5-75.1) | 68.5 (65.5-71.5) | 34.1 (30.1-38.2) | N/A | 88.8 (86.4- 91.2) | 84.7 (82.1- 87.4) | 61.9 (50.6-73.1) | 62.5 (59.3-65.7) | 80.9 (76.7-85.1) | N/A | N/A | 70.2 (68.2-71.1) | [63] |
|  | HD | N/A | N/A | N/A | N/A | N/A | N/A | N/A | N/A | N/A | N/A | N/A | N/A |  |
|  | PD | N/A | N/A | N/A | N/A | N/A | N/A | N/A | N/A | N/A | N/A | N/A | N/A |  |
|  | KTx | N/A | N/A | N/A | N/A | N/A | N/A | N/A | N/A | N/A | N/A | N/A | N/A |  |
| Portugal | ESRD | N/A | N/A | N/A | N/A | N/A | N/A | N/A | N/A | N/A | N/A | N/A | N/A | [82] |
|  | HD | 76.10± 16.46 | 66.52± 21.05 | 25.14± 24.49 | 12.54± 28.38 | 77.8± 20.0 | 81.4± 19.4 | 78.0± 26.8 | 40.1± 15.3 | 81.2± 28.5 | 89.5± 20.8 | 59.4± 23.2 | N/A |  |
|  | PD | N/A | N/A | N/A | N/A | N/A | N/A | N/A | N/A | N/A | N/A | N/A | N/A |  |
|  | KTx | N/A | N/A | N/A | N/A | N/A | N/A | N/A | N/A | N/A | N/A | N/A | N/A |  |
| Spain | ESRD | N/A | N/A | N/A | N/A | N/A | N/A | N/A | N/A | N/A | N/A | N/A | N/A | [85] |
|  | HD | 68.0± 20.5 | 55.6± 23.4 | 14.3± 9.9 | 20.2± 28.6 | 30.1± 24.9 | 28.0± 24.2 | 49.2± 39.3 | 53.7± 22.1 | 74.9± 21.8 | 89.2± 14.4 | N/A | N/A |  |
|  | PD | 71.1± 16.6 | 57.6± 24.8 | 17.5± 9.0 | 29.7± 37.8 | 31.5± 30.4 | 28.3± 26.0 | 55.6± 34.3 | 57.8± 23.2 | 76.0± 23.9 | 95.8± 9.5 | N/A | N/A |  |
|  | KTx | N/A | N/A | N/A | N/A | N/A | N/A | N/A | N/A | N/A | N/A | N/A | N/A |  |
| UK | ESRD | 78.5± 15.4 | 79.8± 20.8 | 73.6± 28.5 | 51.4± 38.7 | 85.6± 16.8 | 82.0± 15.9 | 14.7± 30.4 | 57.9± 21.7 | 77.8± 31.9 | N/A | N/A | N/A | [66] |
|  | HD | N/A | N/A | N/A | N/A | N/A | N/A | N/A | N/A | N/A | N/A | N/A | N/A |  |
|  | PD | N/A | N/A | N/A | N/A | N/A | N/A | N/A | N/A | N/A | N/A | N/A | N/A |  |
|  | KTx | N/A | N/A | N/A | N/A | N/A | N/A | N/A | N/A | N/A | N/A | N/A | N/A |  |
| US | ESRD | N/A | N/A | N/A | N/A | N/A | N/A | N/A | N/A | N/A | N/A | N/A | N/A | [70] |
|  | HD | N/A | N/A | N/A | N/A | N/A | N/A | N/A | N/A | N/A | N/A | N/A | N/A |  |
|  | PD | N/A | N/A | N/A | N/A | N/A | N/A | N/A | N/A | N/A | N/A | N/A | N/A |  |
|  | KTx | 80.5± 14.0 | 71.2± 21.3 | 50.9± 28.8 | N/A | N/A | N/A | N/A | N/A | N/A | N/A | N/A | N/A |  |

For each scale assessed, item scores were summed and transformed using a scale of zero-100, with higher scores showing better HRQoL. Abbreviations: Symp= Symptoms; EKD= Effects of kidney disease; BKD= Burden of kidney disease; Cogn= Cognitive function; QSI= Quality of social interaction; Sex= Sexual function; Soc= Social support; SE= Staff encouragement; PS= Patient satisfaction; ESRD= end-stage renal disease; SD= standard deviation; CI= confidence interval; RRT= renal replacement therapy; HD= haemodialysis; PD= peritoneal dialysis; KTx= kidney transplantation; N/A= non-available.
